# Utilization of a Pediatric DCD Liver Following Normothermic Regional Perfusion: A Case Report on the Youngest Donor in the United States

**DOI:** 10.1111/petr.70198

**Published:** 2025-10-02

**Authors:** Raphaël M. J. Fischer, Ahmad Abdelaal, Ashley Sweet, Nicolas Muñoz, Peter L. Abt, Samir Abu‐Gazala

**Affiliations:** ^1^ Department of Surgery, Perelman School of Medicine University of Pennsylvania Philadelphia Pennsylvania USA; ^2^ Life Connection of Ohio Kettering Ohio USA

**Keywords:** donation after circulatory death, liver, normothermic regional perfusion, transplantation

## Abstract

**Background:**

Normothermic regional perfusion (NRP) is gaining rapid popularity in adult donation after circulatory death (DCD) to increase organ utilization and improve outcomes. However, literature is lacking for the pediatric population. We therefore present the youngest DCD donor in the United States from whom a liver was recovered with NRP and subsequently transplanted.

**Methods:**

The donor was a 5‐year‐old male who underwent thoraco‐abdominal NRP for kidney and liver procurement. In total, 72 min passed from the withdrawal of life‐sustaining treatment to the start of NRP, resulting in 10 min of functional warm ischemia time. The donor was perfused for 80 min, with lactate levels decreasing from 8.29 at the start of perfusion to 5.40 mmol/L at the end of perfusion. The procured graft weighed 480 g and was subsequently transplanted in an adult female recipient with decompensated cirrhosis due to alcohol‐associated liver disease.

**Results:**

The liver was successfully utilized and functioned immediately with no graft‐specific complications. The patient was discharged on postoperative day 39.

**Conclusions:**

This case demonstrates that NRP can be applied effectively in small pediatric donors, yielding excellent early graft function. Our experience adds to the emerging literature on pediatric NRP. We conclude that broader adoption of NRP could help increase the donor pool and ease the strain on the pediatric waiting list.

**Abbreviations**
A‐NRP: abdominal normothermic regional perfusion
BMI: body mass index
DCD: donation after cardiac death
fWIT: functional warm ischemia time
HOPE: hypothermic machine perfusion
HTK: histidine‐tryptophan‐ketoglutarate
MELD: model for end‐stage liver disease
NRP: normothermic regional perfusion
OPO: organ procurement organization
OPTN: Organ Procurement & Transplant Network
SRR: super‐rapid recovery
TA‐NRP: thoracoabdominal normothermic regional perfusion
TDWIT: total donor warm ischemia time
TIPS: transjugular intrahepatic portosystemic shunt
U.S.: United States
UDDA: united states uniform determination of death act
WLST: withdrawal of life sustaining treatment

## 1 Introduction

Over the past 5 years, the use of machine perfusion technologies including normothermic regional perfusion (NRP) in donation after circulatory death (DCD) organ procurement has dramatically changed the landscape of liver transplantation in the United States [1]. After declaration of death of a DCD donor, rapid aortic and venous cannulation is obtained and initiation of mechanical perfusion of the abdominal organs (A‐NRP) or thoracic and abdominal organs (TA‐NRP) is restored in situ with oxygenated blood at physiological temperature. NRP replenishes depleted energy reserves by reoxygenation, allows for quality assessment of the perfused liver, and reduces time pressure for organ retrieval [2]. In the U.S., implementation of NRP has been shown to increase organ utilization rate and improve outcomes in DCD liver transplantation among adult recipients compared to super rapid‐recovery [3, 4, 5]. However, there is limited data on the use of NRP among pediatric DCD donors.

To date, the youngest reported donor undergoing NRP for liver procurement was a 2.5‐month‐old infant in Spain [6]. Additionally, a study from Spain examined 13 pediatric donors undergoing NRP, of whom 11/13 (85%) of the livers were recovered and 11/11 (100%) were transplanted with no post‐transplant complications [7]. Even without the use of NRP, literature has suggested that rapidly recovered cold‐stored livers from pediatric DCD donors have similar outcomes to grafts recovered from pediatric DBD donors [8, 9]. Despite these favorable results, pediatric DCD livers in the United States are procured at a significantly lower rate than adult DCD livers, particularly among the 0–12‐year‐old age group [8]. Thus far, no U.S. case of liver transplantation following pediatric NRP has been reported. We therefore present the youngest U.S. DCD donor from whom a liver was recovered with NRP and subsequently transplanted.

## 2 Donor and Procurement

The donor was a 5‐year‐old male (18.1 kg; BMI 22.9 kg m^2^) born at 31 weeks' gestation with cerebral ventriculomegaly, hypoxic ischemic encephalopathy, seizure disorder, blindness, hearing impairment, dysphagia with gastrostomy‐tube dependence, hypothyroidism, osteopenia, and perinatal asphyxia with respiratory failure requiring tracheostomy and ventilator dependence who presented with 3 days of progressive hypoxia. Due to the poor prognosis, the decision to proceed with withdrawal of life‐sustaining treatment (WLST) and organ donation was made by the parents in conjunction with the patient's medical team. Prior to obtaining donor authorization from the parents, the Life Connection organ procurement organization (OPO) carefully explained the general donation procedure, as well as the involvement, benefits, and risks of NRP.

**FIGURE 1:**
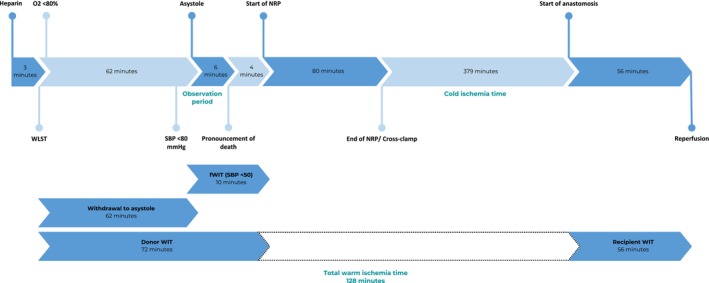
Representation of the donation and transplant procedure. fWIT, functional warm ischemia time; MAP, mean arterial pressure; NRP, normothermic regional perfusion; SBP, systolic blood pressure; WIT, warm ischemia time; WLST, withdrawal of life‐sustaining treatment.

Three minutes before WLST in the operating room, 5400 units (395 units per kilogram) of heparin were given. Asystole was noted 62 min after WLST followed by a 6‐min observation period, after which cardiopulmonary death was confirmed. Subsequently, TA‐NRP was performed with clamping of the aortic arch vessels, followed by cannulation of the ascending aorta (14 French Medtronic DLP Cannula) and right atrium (20 French Medtronic DLP Cannula) for arterial inflow and venous outflow, respectively. NRP was initiated 4 min after incision, resulting in 72 min of total donor warm ischemia time (TDWIT) and a functional warm ischemia time (fWIT) of 10 min, defined as a systolic blood pressure below 50 mmHg, per the Life Connection Organ Procurement Organization. In this donor, the start of fWIT coincided with asystole. To determine if the liver should have been procured, the current maximal waiting time for procurement was 120 min of TDWIT and 30 min of fWIT. Oxygen saturation was not monitored, as the distribution of blood to vital organs may alter the accuracy of the pulse oximeter during the donation process. Reestablishment of cerebral blood flow was not monitored during this case. A representation of the donation procedure is depicted in Figure 1.

**FIGURE 2:**
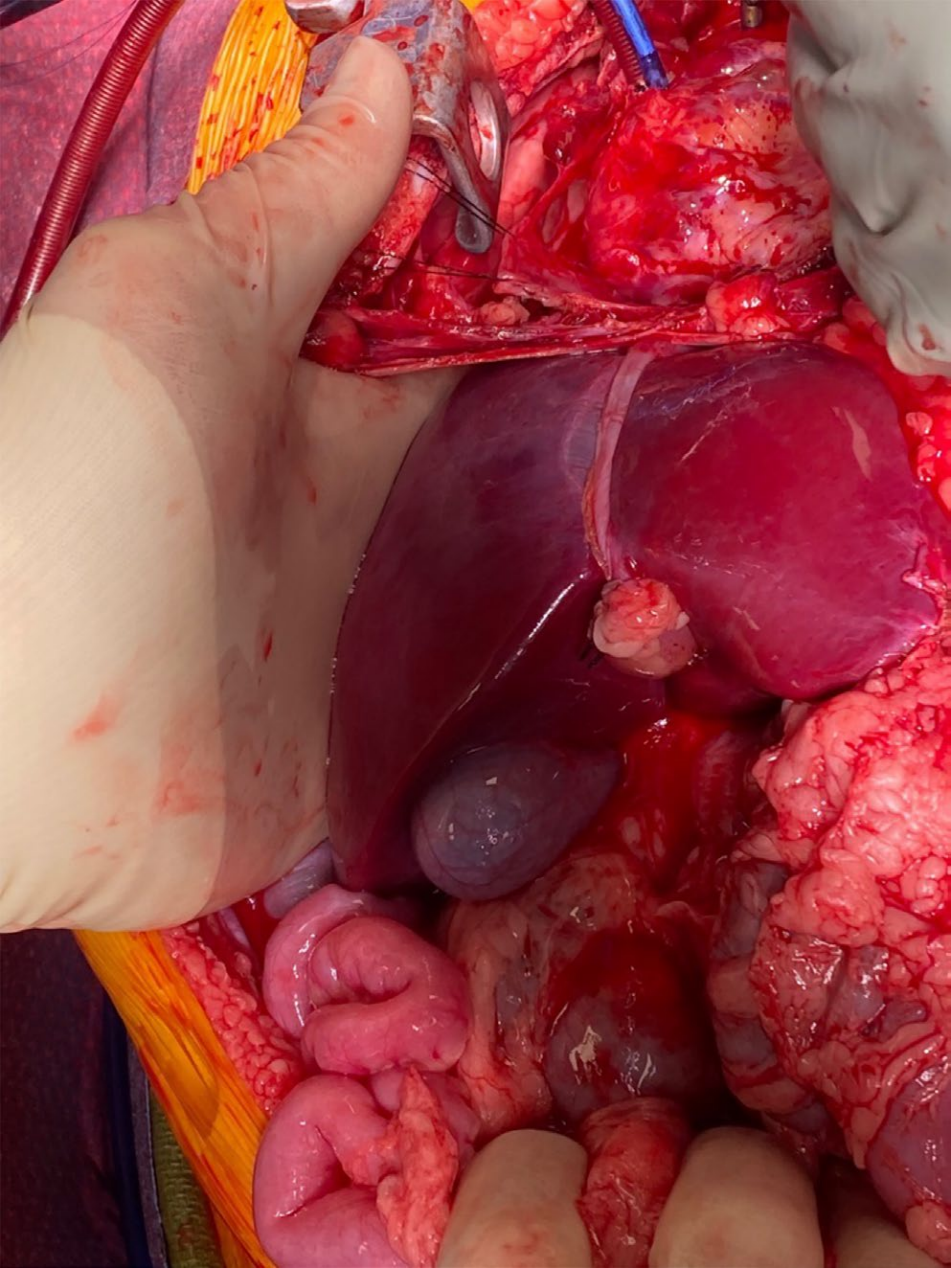
Picture of the liver during normothermic regional perfusion with cannulation of the right atrium and ascending aorta.

The NRP circuit was primed with two units of blood before the start of perfusion, with an additional unit infused 17 min into perfusion, with a goal of maintaining the hematocrit level at or above 20% to ensure adequate oxygen delivery. The average flow rate was 1.9 L/min, ranging from 1.51 to 2.10 L/min. Lactate levels decreased from 8.29 to 5.40 mmol/L from start to end of perfusion. Additional laboratory values are reported in Table 1. Liver congestion was noted shortly after the initiation of NRP, requiring adjustment of the venous cannula. The macroscopic anatomy of the liver is depicted in Figure 2. During perfusion, attention was given to the overall lactate clearance, with preference for at least 2 downward lactate trend results before accepting the liver. Lactate levels were measured every 15–30 min, and no transaminases were assessed. Similarly, to assess liver viability, the common bile duct was dissected, transected, and cannulated with a small sterile Foley catheter, after which bile production was observed. After 80 min of perfusion, it was decided to stop the perfusion based on a persistent downward trend in lactate values. Before cross‐clamping, 2700 units (150 units per kilogram) of heparin were administered. NRP was stopped with simultaneous cross‐clamping of the aortic root and the abdominal aorta at the iliac bifurcation. Three liters of cooled histidine‐tryptophan‐ketoglutarate (HTK) preservation solution were flushed through the aortic‐arch cannula prior to completion of hepatectomy, followed by one‐liter HTK portal flush on the back table. The liver weighed 480 g and was transported on ice to the recipient hospital. Only the liver and kidneys were recovered with intent for transplant. The total time from cold flush to start of anastomosis in the recipient was 379 min, with an additional 56 min of warm ischemia time until simultaneous portal and arterial reperfusion.

**TABLE 1:** Donor laboratory and perfusion values during NRP.

	0′	20′	35′	45′	60′	75′
pH	7.11	7.32	7.66	7.66	—	—
pCO_2_	52.6	41.4	29.4	24.4	—	—
pO_2_	685	670	671	677	—	—
BE	−12	−4	12	7	—	—
HCO_3_	16.8	21.8	33.3	27.7	—	—
Na	145	150	157	—	—	—
K	6.2	4.5	4.4	—	—	—
Ca	0.65	1.20	1.48	—	—	—
Glucose	298	313	299	—	—	—
Lactate	8.29	8.65	7.94	7.54	6.62	5.40
Flow	1.51	2.10	2.00	2.04	1.56	2.10
MAP	62	59	59	58	—	—

Abbreviations: BE, base excess; Ca, calcium (mg/dL); Flow (L/min); Glucose (mg/dL); HCO_3_, bicarbonate (mmol/L); K, potassium (mmol/L); lactate (mmol/L); MAP, mean arterial pressure (mmHg); na, not assessed; Na, sodium (mmol/L); pCO_2_ (mmHg); pO_2_ (mmHg).

## 3 Recipient

The recipient was a 53‐year‐old female with decompensated cirrhosis due to alcohol‐associated liver disease, complicated by ascites requiring large volume paracentesis, hepatic hydrothorax, esophageal varices status post transjugular intrahepatic portosystemic shunt (TIPS), and with hepatopulmonary syndrome. At the time of transplant, the patient had a MELD‐3.0 score of 15, weight of 62.9 kg and BMI of 24.6. She tolerated the procedure well without significant hemodynamic issues on reperfusion. Her graft functioned immediately. Her postoperative course was complicated by prolonged weaning from mechanical ventilation as a result of delirium in the setting of steroids, tacrolimus, and acute kidney injury. On postoperative day 39, she was discharged with normalized liver function tests. The post‐transplant laboratory values are depicted in Table 2. Since the transplantation, no graft‐related complications have occurred.

**TABLE 2:** Recipient post‐transplant laboratory values.

	POD 0	POD 1	POD 2	POD 3	POD 4	POD 5	POD 6	POD 7	POD 39
ALT	90	74	88	92	83	75	57	48	24
AST	274	103	89	82	69	54	54	31	19
AP	46	40	49	57	69	77	77	74	82
Total bilirubin	5.9	7.7	8.1	7.3	7.0	6.7	5.3	4.7	0.5
INR	1.5	1.5	3.3	2.4	1.6	1.3	1.4	1.3	1.1

Abbreviations: ALT, alanine aminotransferase; AP, alkaline phosphatase; AST, aspartate aminotransferase; INR, international normalized ratio; POD, postoperative day.

## 4 Discussion

We describe a successful liver transplant recovered from the youngest reported liver donor undergoing NRP in the U.S. The procurement was uneventful, feasible, and resulted in a fully functioning graft. These are important results, as the global experience with pediatric NRP is limited and the literature is lacking. An analysis of the United Network for Organ Sharing (UNOS) has shown that NRP‐procured pediatric livers had a 100% utilization rate [3]. Additionally, a retrospective cohort study from Spain on NRP‐procured pediatric grafts, reported a 100% acceptance rate of 11 donor livers [7]. These data support the idea that functional assessment of liver grafts during NRP decreases organ discard rates. Additionally, both studies reported excellent recipient outcomes.

This is the first U.S. case of TA‐NRP liver donation from a small pediatric donor, demonstrating feasibility and good short‐term outcomes in an adult recipient. The implementation of NRP in the pediatric DCD donor may be the beginning of an early growth phase of pediatric NRP, mirroring national trends in adult DCD procurement [10, 11]. Importantly, we detail the cannulation technique and perfusion parameters in a small donor, similar to a recent case report from Duke, but highlight the utilization of the liver for transplant [12]. Unlike the Duke experience of pediatric TA‐NRP with the heart transplanted into a pediatric recipient, our case describes the procurement of a pediatric liver transplanted into an adult female recipient of average to small size. Prior literature showed evidence for decreased waitlist mortality among small‐sized women when offered a pediatric liver graft [13, 14].

Pediatric NRP could also benefit pediatric recipients and decrease waitlist mortality. This is important because liver candidates under the age of 1 have the highest waitlist mortality of all age groups [15]. Due to the scarcity of sized matched livers, small pediatric recipients currently rely heavily on adult split‐liver grafts to be transplanted [16]. Furthermore, pediatric recipients of DCD livers have been noted to have better graft and patient survival than adult recipients, further supporting the use of pediatric NRP‐procured liver grafts for pediatric recipients [17]. However, recent data suggested that pediatric DCD livers are more often discarded than adult DCD liver grafts when recovered with SRR, and if accepted, they are primarily allocated to adult recipients [8]. Consequently, NRP offers the possibility of utilizing organs from DCD donors that would otherwise go unused and may have the ability to improve outcomes compared to SRR.

In addition to the use of in situ perfusion techniques, pediatric DCD liver transplant may benefit from the use of ex‐situ perfusion devices to facilitate transplantation of small grafts. For example, a review by Parente et al. reported the feasibility of using hypothermic oxygenated machine perfusion (HOPE) in small pediatric and partial grafts, resulting in low rates of reperfusion injury and excellent outcomes [18]. However, there are no pediatric donors involved in the large randomized controlled trials of normothermic regional perfusion, and data on the ability to perfuse small grafts on normothermic devices are limited [19, 20]. Ex‐situ machine perfusion, in combination with NRP, may work synergistically to increase the graft utilization rate and improve outcomes.

Despite the early positive results with pediatric NRP, there remains ongoing professional and public deliberation surrounding the ethical permissibility of NRP. Firstly, some have argued that the restoration of mechanical circulation via NRP may violate the United States Uniform Determination of Death Act (UDDA), which states that circulatory death can only be declared when there is an irreversible cessation of circulatory and respiratory functions [21]. However, it has been argued that in the context of NRP, it is not circulation per se that is relevant for defining death, but rather circulation restoration to the brain [22]. For this reason, once death has been declared, the arteries originating from the aortic arch are clamped during TA‐NRP, and the descending aorta is clamped during A‐NRP to restrict cerebral circulation. This raises a second concern, that the act of interrupting cerebral blood flow may violate the dead donor rule, an ethical principle that organ donation cannot cause the death of the donor, by precipitating brain death when mechanical circulation is restored [23]. However, as has previously been argued by Wall et al., NRP reestablishes circulation only *after* circulatory death has been declared and a period of hands‐off time has elapsed to ensure there is no autoresuscitation of the donor [24]. Furthermore, one could assert that cerebral circulation has already stopped before the commencement of NRP, and restricting brain perfusion simply ensures it remains abrogated.

An additional physiological concern regarding NRP, which becomes particularly important for the pediatric donor, is whether restoration of regional circulation restores retrograde brain circulation via vertebrobasilar collaterals, and therefore the possibility of consciousness or sensory perception. For this reason, many NRP protocols not only clamp the aortic arch vessels but also vent them to atmospheric pressure in order to shunt any collateral blood flow away from the brain. Studies of adult DCD‐NRP donors have been reassuring that the amount of collateral cerebral flow is inadequate to support neuronal function [25, 26, 27]. However, in the pediatric population, little is known about the degree of autonomic shunting to collaterals and brain resilience after circulatory arrest. Cerebral blood flow is routinely monitored during NRP recoveries in Spain, a practice which could perhaps be instituted more broadly in order to gather more data about cerebral perfusion in pediatric donors [28]. In part due to these concerns, the European Society of Organ Transplantation has yet to endorse pediatric NRP [29].

Finally, attention must also be given to the authorization of donation by families of pediatric donors. In contrast to adult donors, where donation is either first‐person authorized or based on substituted judgment by relatives or friends, the decision in pediatrics is based on the best interest standard. To make an informed decision, clear consensus should be reached on the stance of institutions and OPOs towards what technical aspects of NRP are shared and what uncertainties exist. Currently, significant variation in NRP practice exists among OPOs in the U.S. [10] This poses a challenge among families who may have learned about the opportunity to donate but are denied due to the vagaries of hospital or OPO practice. Transparent and sufficient details should be made available to families considering NRP organ donation, in a digestible and empathetic format. However, the lack of consensus among professionals complicates this, as no uniform guidelines on NRP technique, best practices, or donor family assent are available at this time. These are critical to ensure the safe, ethical, and successful implementation of NRP practices in pediatric donors. Given the previously described concerns, the medical community and the U.S. transplant network continue to have rigorous discussions around the ethics of NRP. A recent white paper published by the Organ Procurement & Transplant Network (OPTN) concluded that it should continue with the implementation of NRP while simultaneously ensuring that appropriate principles and procedures are established, preserving the credibility and trust in the transplant system [30].

In conclusion, we demonstrate that NRP can be applied effectively to recover small pediatric DCD livers, yielding excellent early graft function. Our experience adds to the emerging literature that shows NRP may increase liver utilization rates and improve postoperative outcomes compared with SRR. Furthermore, we discuss important ethical concerns of this evolving practice, including pediatric‐specific considerations, and suggest opportunities to address them. Broader adoption of NRP could help increase the donor pool and ease the strain on the pediatric waiting list.

## Data Availability

Data sharing not applicable to this article as no datasets were generated or analysed during the current study.

## References

[1] A. Wall , A. Gupta , and G. Testa , “Abdominal Normothermic Regional Perfusion in the United States: Current State and Future Directions,” Current Opinion in Organ Transplantation 29, no. 3 (2024): 175–179, 10.1097/MOT.0000000000001144. 38506730

[2] A. S. Shah , “Normothermic Regional Perfusion in Donor Heart Recovery: Establishing a New Normal,” Journal of Thoracic and Cardiovascular Surgery 164, no. 1 (2022): 142–146, 10.1016/j.jtcvs.2021.11.084. 34952705

[3] A. L. Zhou , A. Leng , J. M. Ruck , et al., “Utilization and Outcomes of Abdominal Transplants Using Thoracoabdominal Normothermic Regional Perfusion in Pediatric Donation After Circulatory Death: The United States Experience,” Transplantation 108, no. 7 (2024): e154–e155, 10.1097/TP.0000000000005046. 38917245 PMC11207187

[4] M. T. Sellers , J. Grandas , M. T. Warhoover , J. D. Poland , and D. C. Clapper , “Normothermic Regional Perfusion Performed by a United States Organ Procurement Organization for Nonthoracic Organ Donors,” American Journal of Transplantation 25 (2025): 1677–1684, 10.1016/j.ajt.2025.04.005. 40209904

[5] A. J. Hessheimer , E. Coll , F. Torres , et al., “Normothermic Regional Perfusion vs. Super‐Rapid Recovery in Controlled Donation After Circulatory Death Liver Transplantation,” Journal of Hepatology 70, no. 4 (2019): 658–665, 10.1016/j.jhep.2018.12.013. 30582980

[6] A. M. Andres , J. L. Encinas , A. Sánchez‐Galán , et al., “First Case Report of Multivisceral Transplant From a Deceased Cardiac Death Donor,” American Journal of Transplantation 23, no. 4 (2023): 577–581, 10.1016/j.ajt.2022.12.021. 36725427

[7] E. Miñambres , B. Estébanez , M. Á. Ballesteros , et al., “Normothermic Regional Perfusion in Pediatric Controlled Donation After Circulatory Death Can Lead to Optimal Organ Utilization and Posttransplant Outcomes,” Transplantation 107, no. 3 (2023): 703–708, 10.1097/TP.0000000000004326. 36226852

[8] C. J. Little , A. A. S. Dick , J. D. Perkins , E. K. Hsu , and J. D. Reyes , “Livers From Pediatric Donation After Circulatory Death Donors Represent a Viable and Underutilized Source of Allograft,” Liver Transplantation 26, no. 9 (2020): 1138–1153, 10.1002/lt.25795. 32403205

[9] R. van Rijn , P. E. R. Hoogland , F. Lehner , E. L. W. van Heurn , and R. J. Porte , “Long‐Term Results After Transplantation of Pediatric Liver Grafts From Donation After Circulatory Death Donors,” PLoS One 12, no. 4 (2017): e0175097, 10.1371/journal.pone.0175097. 28426684 PMC5398496

[10] M. T. Sellers , J. L. Philip , A. L. Brubaker , et al., “Normothermic Regional Perfusion Experience of Organ Procurement Organizations in the US,” JAMA Network Open 7, no. 10 (2024): e2440130, 10.1001/jamanetworkopen.2024.40130. 39446328 PMC11581661

[11] A. K. Israni , D. A. Zaun , A. Martinez , et al., “OPTN/SRTR 2023 Annual Data Report: Deceased Organ Donation,” American Journal of Transplantation 25, no. 2S1 (2025): S490–S517, 10.1016/j.ajt.2025.01.026. 39947809 PMC12334191

[12] Z. Beckerman , D. Overbey , B. S. Bryner , et al., “Infant Heart Transplant Following Donation After Circulatory Death Using Normothermic Regional Perfusion and Distant Transport, First Reported Case in North America,” JTCVS Techniques 20 (2023): 156–157, 10.1016/j.xjtc.2023.04.001. 37555051 PMC10405160

[13] J. Ge , R. Gilroy , and J. C. Lai , “Receipt of a Pediatric Liver Offer as the First Offer Reduces Waitlist Mortality for Adult Women,” Hepatology 68, no. 3 (2018): 1101–1110, 10.1002/hep.29906. 29604217 PMC6445636

[14] L. D. Nephew , D. S. Goldberg , J. D. Lewis , P. Abt , M. Bryan , and K. A. Forde , “Exception Points and Body Size Contribute to Gender Disparity in Liver Transplantation,” Clinical Gastroenterology and Hepatology 15, no. 8 (2017): 1286–1293.e2, 10.1016/j.cgh.2017.02.033. 28288834 PMC10423635

[15] A. J. Kwong , W. R. Kim , J. R. Lake , et al., “OPTN/SRTR 2023 Annual Data Report: Liver,” American Journal of Transplantation 25, no. 2S1 (2025): S193–S287, 10.1016/j.ajt.2025.01.022. 39947804 PMC12334193

[16] E. R. Perito , G. Roll , J. L. Dodge , S. Rhee , and J. P. Roberts , “Split Liver Transplantation and Pediatric Waitlist Mortality in the United States: Potential for Improvement,” Transplantation 103, no. 3 (2019): 552–557, 10.1097/TP.0000000000002249. 29684000 PMC6773658

[17] C. S. Hwang , S. Levea , J. R. Parekh , Y. Liang , D. M. Desai , and M. MacConmara , “Should More Donation After Cardiac Death Livers Be Used in Pediatric Transplantation?,” Pediatric Transplantation 23, no. 1 (2019): e13323, 10.1111/petr.13323. 30447034

[18] A. Parente , M. Kasahara , V. E. De Meijer , K. Hashimoto , and A. Schlegel , “Efficiency of Machine Perfusion in Pediatric Liver Transplantation,” Liver Transplantation 30, no. 11 (2024): 1188–1199, 10.1097/LVT.0000000000000381. 38619390 PMC11472901

[19] D. Nasralla , C. C. Coussios , H. Mergental , et al., “A Randomized Trial of Normothermic Preservation in Liver Transplantation,” Nature 557, no. 7703 (2018): 50–56, 10.1038/s41586-018-0047-9. 29670285

[20] J. F. Markmann , M. S. Abouljoud , R. M. Ghobrial , et al., “Impact of Portable Normothermic Blood‐Based Machine Perfusion on Outcomes of Liver Transplant: The OCS Liver PROTECT Randomized Clinical Trial,” JAMA Surgery 157, no. 3 (2022): 189–198, 10.1001/jamasurg.2021.6781. 34985503 PMC8733869

[21] “Guidelines for the Determination of Death. Report of the Medical Consultants on the Diagnosis of Death to the President's Commission for the Study of Ethical Problems in Medicine and Biomedical and Behavioral Research,” JAMA 246, no. 19 (1981): 2184–2186. 7289009

[22] J. L. Bernat , B. Domínguez‐Gil , A. K. Glazier , et al., “Understanding the Brain‐Based Determination of Death When Organ Recovery Is Performed With DCDD In Situ Normothermic Regional Perfusion,” Transplantation 107, no. 8 (2023): 1650–1654, 10.1097/TP.0000000000004642. 37170405

[23] R. D. Truog , F. G. Miller , and S. D. Halpern , “The Dead‐Donor Rule and the Future of Organ Donation,” New England Journal of Medicine 369, no. 14 (2013): 1287–1289, 10.1056/NEJMp1307220. 24088088

[24] A. E. Wall , A. Fiedler , S. Karp , A. Shah , and G. Testa , “Applying the Ethical Framework for Donation After Circulatory Death to Thoracic Normothermic Regional Perfusion Procedures,” American Journal of Transplantation 22, no. 5 (2022): 1311–1315, 10.1111/ajt.16959. 35040263

[25] F. F. Dalsgaard , N. Moeslund , Z. L. Zhang , et al., “Clamping of the Aortic Arch Vessels During Normothermic Regional Perfusion After Circulatory Death Prevents the Return of Brain Activity in a Porcine Model,” Transplantation 106, no. 9 (2022): 1763–1769, 10.1097/TP.0000000000004047. 35066546

[26] M. Royo‐Villanova , E. Miñambres , J. M. Sánchez , et al., “Maintaining the Permanence Principle of Death During Normothermic Regional Perfusion in Controlled Donation After the Circulatory Determination of Death: Results of a Prospective Clinical Study,” American Journal of Transplantation 24, no. 2 (2024): 213–221, 10.1016/j.ajt.2023.09.008. 37739346

[27] J. A. Frontera , A. Lewis , L. James , et al., “Thoracoabdominal Normothermic Regional Perfusion in Donation After Circulatory Death Does Not Restore Brain Blood Flow,” Journal of Heart and Lung Transplantation 42, no. 9 (2023): 1161–1165, 10.1016/j.healun.2023.05.010. 37211334

[28] J. C. L. Sergio , “Protocolo Nacional de Transplante Cardiaco de Donante en Asistolia Controlada,” (2023), https://www.ont.es/wp‐content/uploads/2023/08/Protocolo‐Nacional‐de‐Trasplante‐Cardiaco‐de‐Donacion‐en‐Asistolia‐Controlada_Actualizacion‐Marzo‐2023.pdf.

[29] J. Brierley , A. Pérez‐Blanco , J. Stojanovic , et al., “Normothermic Regional Perfusion in Paediatric Donation After Circulatory Determination of Death‐The Oxford Position Statement From ELPAT,” Frontiers in Transplantation 3 (2024): 1320783, 10.3389/frtra.2024.1320783. 38993761 PMC11235291

[30] OPTN Ethics Committee , “Ethical Analysis of Normothermicregional Perfusion (NRP),” (2023), https://optn.transplant.hrsa.gov/media/unfj5nvq/ethical‐implications‐of‐normothermic‐regional‐perfusion_pcsummer2023.pdf.

